# Exploring the Host Range of *Rose rosette Virus* among Herbaceous Annual Plants

**DOI:** 10.3390/pathogens11121514

**Published:** 2022-12-10

**Authors:** Osama O. Atallah, Sherin M. Yassin, Natalie Shirley, Jeanmarie Verchot

**Affiliations:** 1 Department of Plant Pathology & Microbiology, Texas A&M University, College Station, TX 77845, USA; 2 Department of Plant Pathology, Faculty of Agriculture, Zagazig University, Zagazig 44519, Egypt; 3 Plant Pathology Research Institute, Agriculture Research Center, Giza 12619, Egypt

**Keywords:** Emaravirus, *Rose rosette virus*, host range, negative strand RNA virus, infectious clone, rose, RT-PCR

## Abstract

To study the host range of *Rose rosette virus* (RRV), we employed crude sap inoculum extracted from RRV-infected roses and the RRV infectious clone. We inoculated plants from the families *Solanaceae*, *Cucurbitaceae*, *Leguminosae*, *Malvaceae*, *Amaranthaceae*, and *Brassicaceae.* Reverse transcription-polymerase chain reaction (RT-PCR) was used to detect RRV in the inoculated plants throughout their growth stages. Interestingly, RRV was detected in the newly developed leaves of tomato, pepper, tobacco, cucumber, squash, zucchini, pumpkin, pea, peanut, soybean, spinach, okra, and *Chenopodium* spp. The speed of upward advancement of RRV within infected plants was variable between plants as it took two to three weeks for some plant species and up to five weeks in other plant species to emerge in the newest leaves. No severe symptoms were detected on most of the inoculated plants. *Chenopodium* spp., spinach, cucumber and *Nicotiana rustica* exhibited either chlorotic or necrotic lesions with variable shapes and patterns on the systemically infected leaves. Double membrane-bound particles of 80–120 nm in diameter were detected by transmission electron microscopy in the infected tissues of cucumber, pepper, and *N. benthamiana* plants. This finding infers the validity of mechanical inoculation for RRV on a wide range of plants that would serve as potential natural reservoirs.

## 1. Introduction

Rose rosette disease (RRD) is one of the greatest threats to rose cultivation throughout the United States (USA) and North America [1,2], causing significant losses estimated in the millions of dollars [3]. RRD symptoms include rapid stem elongation, extensive thorniness, red pigmentation on new shoots, leaf deformation, witches’ broom, and eventual plant death [1,2]. The first description of RRD was by Conners (1941) [4], and the causal agent, *Rose rosette virus* (RRV), was identified in 2011 [5]. The National Plant Diagnostic Network-Rose (NPDN-R) employs a standard reverse transcription (RT)-PCR test for monitoring the occurrence of RRV infection [6,7]. Roserosette.org is a website hosted by the Center for Invasive Species and Ecosystem Health at the University of Georgia to provide information and enable RRD reporting by the public and scientists [8]. Roserosette.org also provides a North American distribution map to track the geographic spread of RRD based on reported and confirmed incidences [5,9]. 

RRV, a member of the family *Fimoviridae* and the genus *Emaravirus*, has seven RNA genome segments in the negative sense orientation [5,9,10]. Its virions consist of a double membrane envelope surrounding seven ribonucleoprotein complexes comprising all RNA segments. These amorphous virions are generally 120–150 nm in diameter [10,11,12]. The genome segments RNA1 through RNA7 are monocistronic except RNA6, which encodes two overlapping open reading frames. The genomic segments encode at least one open reading frame flanked by noncoding regions. RNA1 encodes the putative viral replicase (RdRp), RNA2 encodes the putative glycoprotein (Gly) precursors, RNA3 encodes the putative viral nucleocapsid (N) protein, and RNA4 encodes the potential viral movement protein (MP). The functions of viral proteins encoded by RNAs 5, 6, and 7 have yet to be characterized. We recently developed an infectious cDNA clone of RRV [13,14] to study virus–host interactions and viral gene functions. 

The vector for RRV is a wind-borne eriophyid mite, *Phyllocoptes fructiphilus* [15,16] similar to other emaraviruses [17,18]. Koch’s postulates of RRD were fulfilled [9] via eriophyid mites, indicating that RRV is the sole causal agent for RRD and confirming the role of eriophyid mites in disease transmission. The virus can also be vectored via infected plant propagation material [19], shoot grafting, and mechanical inoculation [12,19,20]. RRV was reported to be mechanically transmitted to multiflora roses (*Rosa multiflora*) with very low efficiency [11]. Several prior reports suggested that RRV does not infect herbaceous hosts or indicator plants [19,20,21], and is not transmissible by root grafting, dodder, or infested soil [19,22].

Attempts to study the host range of RRV outside of the genus *Rosa* have been mostly unsuccessful. Foliar symptoms or viral sequences were not associated with the inoculated indicator plants and alternative hosts [12,21,23,24]. Because we reported RRV infection of *N. benthamiana* plants using an infectious clone of RRV or sap inoculum from an RRV-infected rose plant [13,14], we decided to revisit the question of whether RRV can be mechanically transmitted to alternative host plants. In this report, we examined the host range of RRV using commercially available species from *Solanaceae*, *Cucurbitaceae*, *Leguminosae*, *Amaranthaceae*, *Malvaceae*, and *Brassicaceae.* We compared RRV infection using sap inoculum with the Agrobacterium-infiltrated infectious cDNA clones in some plant species. Virions were recovered from systemically infected plants and visualized by transmission electron microscopy. This research sheds new light on potential alternative hosts for RRV and will facilitate more in-depth studies of RRV via mechanical inoculation. 

## 2. Materials and Methods

### 2.1. Plant Materials and RRV-Containing Sap Inoculum

Table 1 lists the herbaceous plant species and taxonomic families used in this study. We obtained seeds from Eden Brothers (Arden, NC, USA) and a local market in College Station, TX, USA. We germinated seeds in 20 cm pots filled with Jolly Gardener^®^ Pro-line C/25 growing mix amended with Osmocote^®^ slow-release fertilizer (20:20:20) and kept in a growth chamber (23 ± 2 °C).

RRV-infected adult rose shrubs (cvs. Ducher and Julia Child), provided by Dr. Kevin Ong (Texas A&M University Plant Disease Diagnostic Laboratory), showing typical RRD were used to prepare crude sap inoculum. Young leaves were homogenized (1:1 *w*/*v*) in phosphate-buffered (10 mm Na_2_HPO_4_, and 1.8 mm KH_2_PO_4,_ pH 7.3). The homogenate was filtered through double-layered cheesecloth, aliquoted into 5-mL tubes, and supplemented with 1 U/mL Applied Biosystems™ RNase inhibitor (Thermo Fisher Scientific Co., Pittsburgh, PA, USA) plus 0.1% (*v*:*v*) Silwet-L77. The crude sap was confirmed to be RRV-positive by reverse transcription-polymerase chain reaction (RT-PCR) primer set number one (Table 2) and then stored at −80 °C until further use. Upon thawing, the crude sap was diluted 10-fold in phosphate buffer and used to mechanically inoculate young plants at the stage of three true leaves. The leaves were lightly dusted with carborundum 400 grit and then rub-inoculated with one ml of sap per plant. The inoculated plants were lightly sprayed with tap water, kept in a shaded area overnight, and then transferred to the greenhouse.

### 2.2. RRV Infectious Clone & Agrobacterium Infiltration

The RRV infectious clone consists of seven cDNAs encoding the segments in the antigenomic orientation. The cDNAs are separately inserted between a duplicated cauliflower mosaic virus (CaMV) 35S promoter and nopaline synthetase (NOS) terminator in pCB301-pXT1 plasmid [13,14]. The RRV infectious clone constructs were transformed in *Agrobacterium tumefaciens* strain GV3101 by electroporation using the GenePulser Xcell™ equipped with PC and CE modules (Bio-Rad Laboratories, Hercules, CA, USA). Transformed *Agrobacterium* cells were selected on yeast extract peptone (YEB) agar plates amended with 50 µg mL^−1^ of kanamycin and rifampicin. Selected colonies were grown overnight in YEB liquid media supplemented with 50 µg mL^−1^ kanamycin and rifampicin, 10 mM MES hydrate (pH 5.85), and 20 µM acetosyringone. The cultures were collected by centrifugation at 5000× *g* for 15 min and resuspended in infiltration buffer (pH 5.6) consisting of 10 mM MgCl_2_, 10 mM MES, and 150 µM acetosyringone. The *A. tumefaciens* cultures were adjusted to OD_600_ 1.0 using the SpectraMax^®^ Quick Drop™ spectrophotometer (Molecular Devices, San Jose, CA, USA), and incubated at room temperature for three hours in the dark. Equal culture volumes were mixed at the dilution of OD_600_ 0.1 and infiltrated to the abaxial surface of plant leaves using a needle-less 1 mL syringe. 

### 2.3. Sampling and Nucleic Acid Extraction 

Leaf disks (1 cm diameter) were collected between two- and five-weeks post inoculation and kept on ice. Total RNA was extracted using the Maxwell 16 RSC plant RNA tissue kit (Promega, Madison, WI, USA) according to the manufacturer’s recommendations. RNA concentrations were evaluated using the NanoDrop™ 2000 spectrophotometer (Thermo Fisher Scientific, Waltham, MA. USA). RNA integrity was examined on an ethidium bromide-stained 1% agarose gel in 1X TAE buffer. Fifty microliters of RNA samples were treated with RNase-free DNase I (VWR, Radnor, PA, USA) and incubated at 37 °C for 10 min. 

### 2.4. Reverse Transcription-Polymerase Chain Reaction (RT-PCR)

Sap inoculum and RNA samples from infected plants were used as templates for cDNA synthesis using the Maxima Reverse Transcription kit according to the manufacturer’s protocol (Thermo Fisher Scientific, Waltham, MA, USA). Two-step RT-PCR was performed using random hexamers for cDNA synthesis, four sets of RRV-specific primers shown in (Table 2), and GoTaq^®^ Green Master Mix (Promega, Madison, WI, USA). Forty PCR cycles were carried out following the initial denaturation at 94 °C for 5 min: denaturation at 94 °C for 30 s, annealing at 60 °C for 20 s, and elongation at 72 °C for 20 s. The final cycle was followed by an extension at 72 °C for 10 min. The PCR amplicons were examined on 2% TBE agarose gels stained with ethidium bromide, and photographed with a ChemiDoc MP imaging system (Bio-Rad Corp, Hercules, CA, USA). PCR amplicons were purified using the Gel/PCR DNA fragment extraction kit (IBI Scientific, Peosta, IA, USA) and sent to Eton Bioscience (San Diego, CA, USA) for sequencing. 

### 2.5. Photography and Compiling Images

Symptoms on plant leaves inoculated with sap or infectious clone were photographed using a Nikon D3400 digital camera equipped with a Nikkor 18–55 mm zoom lens. Electron microscope images were obtained using a Veleta (2 k × 2 k) CCD side mount camera (EMSIS, Münster, Germany). Adobe^®^ Photoshop 2022 (San Jose, CA, USA) was used to compile images (San Jose, CA, USA). 

### 2.6. Contagious Sap Extraction and Transmission Electron Microscopy

Samples of systemically infected leaves was prepared by homogenization in 40 mM KH_2_PO_4_ buffer (pH 7.4) (1:10 *w:v*). Ten microliters of crude virion extracts were placed on the 400-mesh copper grids (formvar/carbon Square Mesh, UB, Electron Microscopy Sciences, USA) for negative contrast electron microscopy, and left to settle at room temperature for 5 min. Grids were stained using 1% uranyl acetate for 30 s and then air-dried. Samples were examined using a Hitachi H-7000 transmission electron microscope (Hitachi High Technologies, Tokyo, Japan) located at the Texas A&M University Microscopy and Imaging Center Core Facility (College Station, TX, USA). 

### 2.7. Bioinformatics Analysis

PCR products were sequenced by Eton Biosciences (San Diego, CA, USA). Sequences of PCR amplicons were pairwise aligned against the reference genome sequence (GenBank ID: NC_015300.1) using the pairwise alignment tool using MEGAX [24]. 

## 3. Results

### 3.1. RT-PCR Detection of RRV in Systemically Infected Plants 

**Figure 1:**
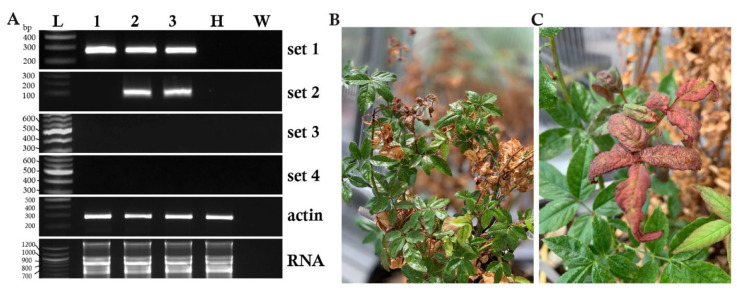
PCR detection of *Rose rosette virus* in infected roses. (**A**) Images of PCR products and total RNA in ethidium bromide-stained 1% agarose gels. The PCR product sizes from primer sets 1 to 4 (listed on the right) are 271, 104, 551, and 500 bp, respectively. Lane L, 100 bp ladder; lane 1, frozen inoculum (cv. Julia Child); lanes 2 and 3, RRV infected rose (cv. Ducher); H, healthy rose leaves; W, nanopure water with no template for RT-PCR. Band sizes of a 100 bp ladder are on the left. (**B**,**C**) This infected rose (cv. Ducher) was used to prepare fresh inoculum and for positive control RNA for RT-PCR tests in subsequent experiments. Typical RRD symptoms (red terminal leaves) are shown.

We tested four primer sets in Table 2 to identify the most reliable to use in this host range study (Figure 1A). The set 1 (RRVF/R) primers [23] are preferred by the National Clean Plant Network-Rose (NCPN-R) for diagnostic detection of RRV [7,8,26,27]. The set 2 (RRV-2F/2R) primers were reported for diagnostic detection of RRV in rose plants and require the addition of PCR amplification facilitators to reduce false negative outcomes [25]. The Verchot laboratory used primer set 3 (RRV3-F2/RRV30917R) and set 4 (agRRV4-F1/R1) for reverse transcription RNAse protection assays (RT-RPA) to detect double-stranded viral RNA in *N. benthamiana* plants infected with the RRV infectious clone [13]. 

Total RNA was extracted from a frozen leaf homogenate obtained from a highly infected RRV plant (cv. Julia Child) [13,14]. We also extracted total RNA from two branches of a highly diseased, RRV- infected rose shrub (cv. Ducher) that we maintain in the greenhouse (Figure 1B,C). RT-PCR was performed, and the set 1 primers confirmed the presence of RRV in all samples. Set 2 primers only detected RRV in the samples from the greenhouse-grown rose shrub. Sets 3 and 4 produced negative results (Figure 1A). Based on these results, we performed subsequent experiments using primer set 1 and the sap from the infected rose plants as a positive control for RT-PCR analyses.

**Figure 2:**
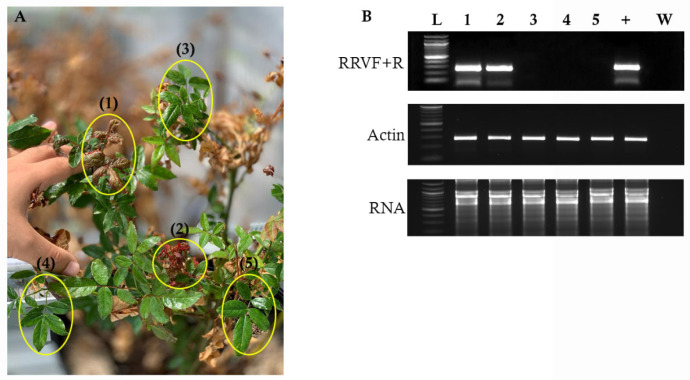
(**A**) The RRV-infected rose plant shows terminal red and green tissues on neighboring branches. Yellow circles identify the harvested leaves for RNA extraction, and the numbers correspond to the lane numbers above the ethidium bromide-stained agarose gel (**B**). The plant shown here is also in Figure 1. Lane L, 100 bp ladder; “+”, healthy rose leaves; W, nanopure water with no template for RT-PCR.

To determine if the RRV-infected rose plant (cv. Ducher) that was an inoculum source is uniformly infected, we extracted total RNA from five branches of the same cane (Figure 2). RT-PCR was performed using the set 1 primers, and the results indicate that only the terminal tissues that showed disease symptoms tested positive for RRV infection. The green tissues did not produce positive RT-PCR results. These data suggested that multiple sampling of terminal tissues may be necessary to detect RRV infection. 

### 3.2. Testing the Susceptibility of Seventeen Plant Species to RRV Infection

**Figure 3:**
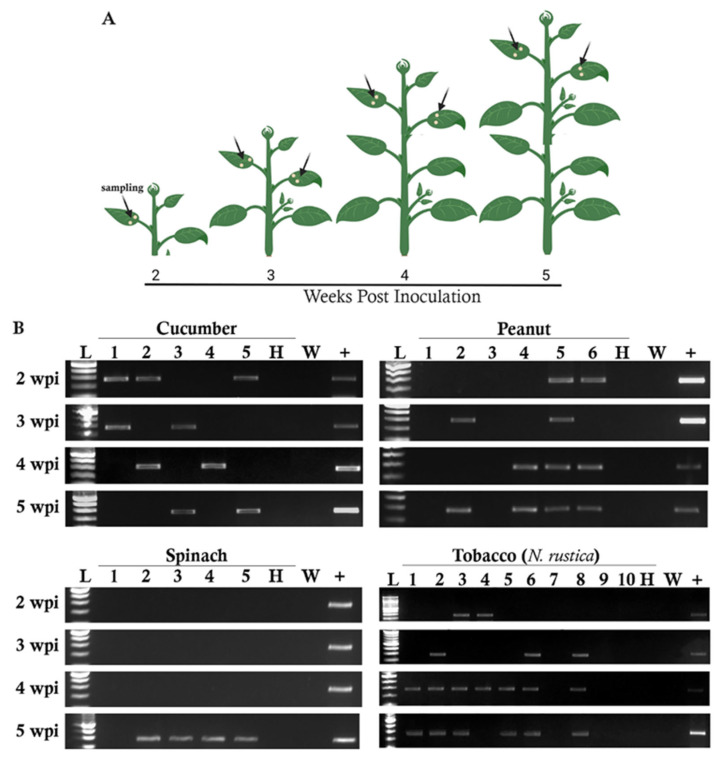
RT-PCR products amplified from the weekly sampling of plants inoculated with RRV-containing crude sap. These representative examples are also in Table 3. (**A**) Diagram showing samples taken between 2 and 5 wpi from newly emerging leaves for RT-PCR testing for RRV infection. (**B**) 271 bp RT-PCR amplicons were analyzed using ethidium bromide-stained 2% agarose gel. The plant’s common names atop each set of gels. The time of sample harvest is indicated on the left. Lane L, 100 bp ladder; lanes 1 through 5, 1 through 6, or 1 through 10 identify individual plants. H represents the mock-inoculated control, W is the water control for the RT-PCR reaction, and “+” identifies the RT-PCR products derived from the sap inoculum.

Seventeen herbaceous plant species were inoculated with crude sap from infected rose shrubs to test their susceptibility to RRV infection (Table 3). Selected plant species belonging to *Cucurbitaceae*, *Solanaceae*, *Leguminosae*, *Amaranthaceae* (with *Chenopodium* species belonging to the subfamily *Chenopodiaceae*), *Malvaceae*, and *Brassicaceae* were inoculated and maintained in the greenhouse for five weeks. Five to ten plants were inoculated with RRV-containing sap, and three plants were treated with phosphate buffer (mock) for each species. We harvested leaf disks from newly emerging leaves each week from 2 to 5 weeks post-inoculation (wpi) (Figure 3A). RT-PCR amplicons were obtained at 2 wpi from the terminal non-inoculated leaves of plants belonging to 10 species (Table 3, Figure 3B). Surprisingly, only seven species produced positive RT-PCR amplicons at 3 wpi, indicating that the newest leaves of some plants did not show evidence of RRV infection (Table 3). Examples of RT-PCR amplicons from cucumber, peanut, and tobacco (*N. rustica*), demonstrate that some plants testing positive for RRV at 2 or 3 wpi can then show negative or positive RT-PCR results in subsequent weeks (Figure 3B). Three cucumber plants tested positive for RRV at 2 wpi, whereas two plants tested positive at 3, 4, and 5 wpi for a total of 5 plants testing positive for RRV during the experiment (Table 3). A total of five winter squash plants tested positive for RRV, of which three plants tested positive at 4 wpi and four tested positive at 5 wpi. One winter squash plant was positive at 4 wpi but negative at 5 wpi. One yellow squash and two *N. glutinosa* plants tested positive for RRV at 2 wpi, and the new leaves sampled between 3 and 5 wpi produced negative results (Table 3). Two tomato and two pea plants tested positive at 2 wpi, and these numbers declined to zero at 4 and 5 wpi. We speculated that these plants might grow faster than the virus can spread into the newly emerging leaves. Only lupine and Arabidopsis did not appear to be susceptible to RRV infection (Table 3). 

### 3.3. Comparing the Infectious Clone and Crude Sap Inoculum for Achieving Systemic RRV Infection

**Figure 4:**
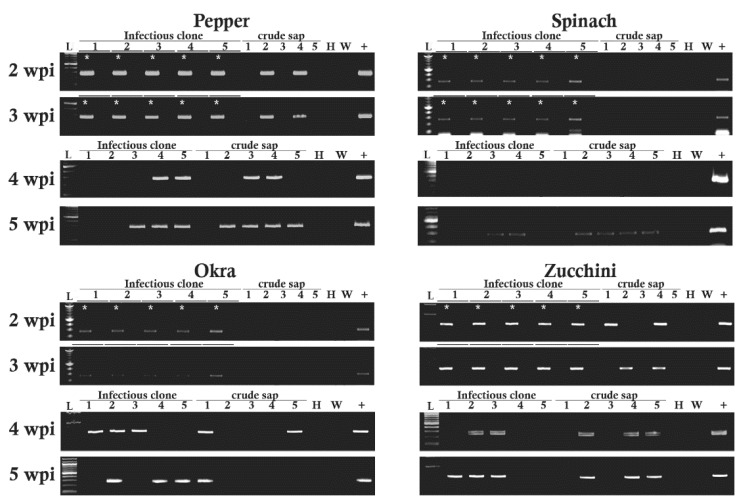
RT-PCR results demonstrating RRV infection of various plants. Detection of RRV at the terminal leaves recorded at 2, 3, 4, and 5 wpi. At 2 and 3 wpi the inoculated (*) and terminal leaves of plants. These plants were inoculated either with the RRV infectious clone or with RRV-containing crude sap. Lane L, 100 bp ladder. Plants numbered 1 through 5, as indicated above the lanes in the ethidium bromide-stained 2% agarose gels. H indicates the mock-inoculated controls, W indicates the water control replacing RNA in the reaction, and + indicates the RT-PCR control using RNA extracted from the RRV sap inoculum.

For comparison, we inoculated *N. benthamiana*, pepper, spinach, okra, and zucchini plants with the RRV infectious clone [13,14] or RRV-containing sap (Table 4 and Figure 4). Then, RNA was extracted from samples taken from the inoculated (local) and upper non-inoculated (systemic) leaves at weekly intervals until 5 wpi (Table 4). At 2 and 3 wpi, inoculated leaves tested positive for RRV infection when the infectious clone was used as the inoculum, and none of the upper leaves were positive for RRV. We detected 2 or 3 systemically infected plants at 4 and/or 5 wpi among the five plant species tested (Table 4 and Figure 4). 

When RRV-containing crude sap was the inoculum, there were two pepper and two zucchini plants systemically infected at 2 wpi. At 3 wpi two zucchini plants also tested positive by RT-PCR but one of these plants was different from the prior week’s testing results. At 4 and 5 wpi, there were four pepper and three zucchini plants that tested positive by RT-PCR for RRV infection. For spinach, systemic infection was first detected at 5 wpi when crude sap was used as the inoculum. For okra, two plants showed systemic infection at 4 wpi (Table 4 and Figure 3). Spinach plants remained healthy for the first four weeks and become infected at 5 wpi. To further assess whether RRV spreads slowly to the upper leaves, we inoculated a subset of plants with either the infectious clone or RRV-containing plant sap. RT-PCR was performed weekly to monitor the spread of infection, and at 2 wpi the inoculated leaves of all plant species tested positive for RRV infection. Systemic infection was most evident at 4 or 5 wpi across hosts.

### 3.4. Symptoms of RRV-Infected Plants and Virions

**Figure 5:**
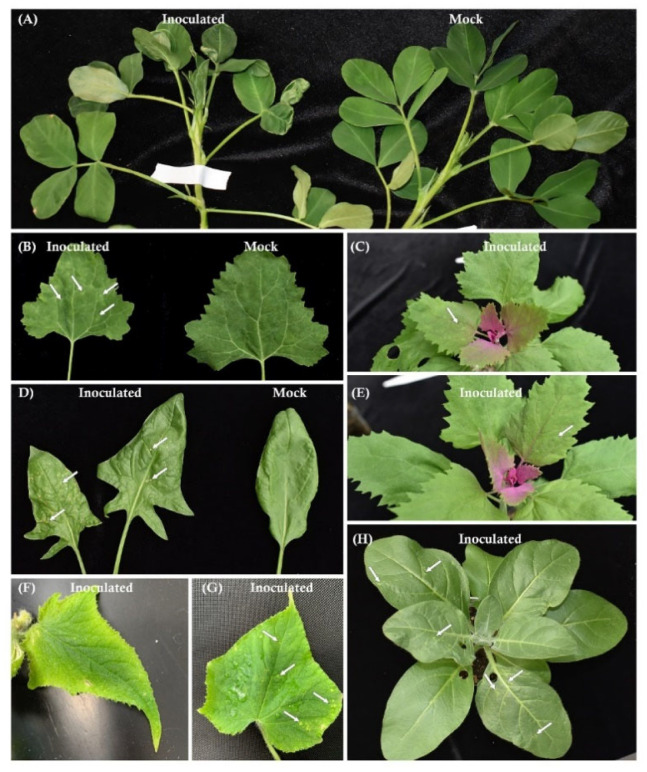
RRV symptoms on various host plants. These plants were mechanically inoculated with contagious crude sap from RRV-infected plants and maintained in the greenhouse. Symptoms developed on inoculated peanut (**A**), *C. quinoa* (**B**), *C. amaranticolor* (**C**,**E**), spinach (**D**), cucumber (**F**,**G**), and *N. rustica* (**H**) plants are shown. Panels (**A**,**B**,**D**) show the healthy on the right and infected plants on the left.

RRV-infected plants generally showed mild or no systemic symptoms until 5 wpi. Only RRV-inoculated peanut plants showed widespread deformation of newly developed leaves between 3 and 5 wpi (Figure 5A). Leaves seemed to be deformed as the result of the arrested development of leaf midribs with expanding leaf blades. RRV-infected leaves generally curled upward. The most common symptoms among RRV-infected *Chenopodium quinoa*, *Chenopodium amaranticolor*, spinach, cucumber, and *Nicotiana rustica* plants were chlorotic foliar lesions appearing within 2 wpi (Figure 5B–H). The shapes and distribution patterns of lesions were slightly different among the infected plant species. Yellow chlorotic lesions occurred on C. quinoa and cucumber leaves (Figure 5B,F,G). Pale green lesions occurred on *C. amaranticolor* and *N. rustica* leaves (Figure 5C,H). In addition, irregular growth (raised areas) of leaf lamina was observed on newly developed *C. amaranticolor* leaves (Figure 5E). The chlorotic lesions on spinach leaves developed into necrotic lesions with time (Figure 5D). These recorded RRV-associated symptoms were reproducible in repeated experiments. 

**Figure 6:**
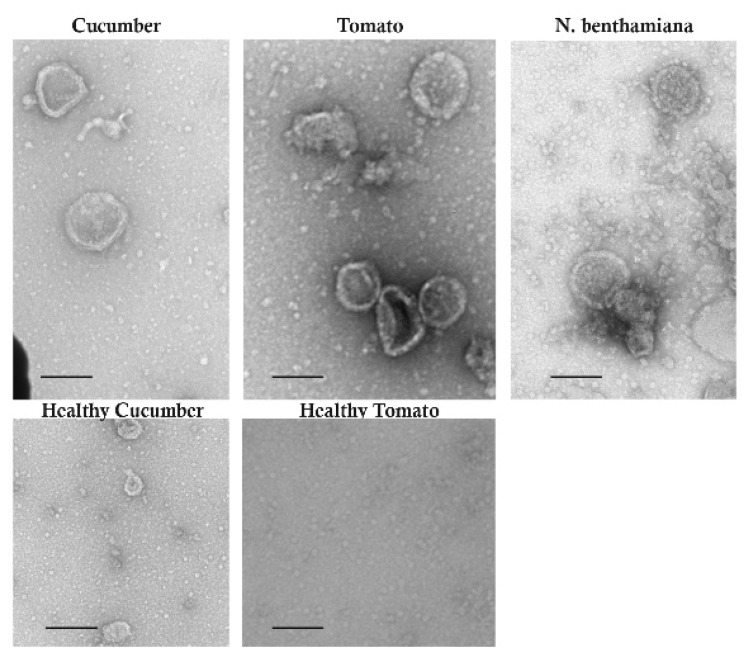
Transmission electron microscopic images of virions in infected leaves. The plant is identified above each image. Black scale bars represent 100 nanometers. The terminal leaves from mock-inoculated plants were selected as the healthy controls.

Leaf extracts from RRV-infected cucumber, tomato, and *N. benthamiana* leaves were spotted onto grids and negative staining was performed for visualization by transmission electron microscopy (Figure 6). We obtained images of semispherical to pleomorphic virions. The measured diameters of these particles were approximately 80–110 nanometers in diameter. No particles were detected in healthy mock-inoculated tissues. 

## 4. Discussion

RRV symptoms in roses vary according to the cultivar, plant developmental stage, and between shooting branches of a bush [28,29,30]. Endpoint PCR and RT-qPCR are preferred for diagnostic detection of RRV infection in roses [7,25,28,31,32], although a few studies reported false negative results [5,13]. Given recent advances in the RT-PCR technology detecting RRV in infected rose leaves, we selected the most reliable RT-PCR primers to re-examine the host range of RRV. This is the first report of RRV infecting plant hosts other than wild and cultivated roses. We mechanically inoculated RRV-containing plant sap and *Agrobacterium*-delivered RRV infectious clone to 17 plant species and identified 15 species as secondary hosts. Prolonged PCR cycling numbers are necessary to obtain RRV-related amplicons and repeated sampling was essential to confirming RRV infection. 

Until the past decade, most reports using Koch’s postulates to investigate RRV transmission to indicator plants have been assessed for visual evidence of disease with few attempts to use molecular diagnostic detection [7,19,26,27]. We employed the most recently developed and widely used diagnostic RT-PCR methods to amplify RRV sequences and to study virus accumulation in mechanically inoculated plants [23]. These primers were recommended for their reliable assessment of RRV infection by the National Rose Rosette Monitoring Network for tracking the geographical spread of RRV [8,29]. Some of the earliest reports of rose rosette disease (RRD) presented attributes of this lethal rose disease, which included symptoms of witch’s broom and malformed flowers, excessive thorniness, and reddened leaves [7,11,23]. Before anyone had isolated the virus agent for RRD it was established that the pathogen was transmitted through eriophyid mites while feeding on infected tissues [30,33]. Grafting experiments were useful for transferring RRD to healthy plants [11]. One study in 1968 prepared sap inoculum from an RRV-infected *R. multiflora* plant to inoculate seedlings of cucumber, squash, and cowpea with negative results. These and other authors concluded that RRV could only infect rose species by grafting or mite transmission [33]. In contrast, mechanical transmission of RRV was reported using microneedles attached to a wooden dowel [27]. In 2001 it was reported that the causal agent of RRD could be mechanically inoculated to *Nicotiana glutinosa* and *N. benthamiana* [26]. These studies occurred before the viral agent had been isolated and, therefore diagnostic detection was dependent upon evidence of disease symptoms, or evidence of typical viral cytopathological structures. Laney (2010) mechanically inoculated 22 indicator plant species with RRV-containing sap from infected rose leaves. RT-PCR was performed for 30 cycles using RNA extracted from plants at 3 wpi and none of the indicator plants tested positive for RRV infection, although the lower detection limits of the PCR primers were not demonstrated [5,21]. Considering the different RT-PCR outcomes obtained using four primer sets in Figure 1, and the outcomes of repeated weekly sampling, it is likely that the approach used by Laney (2010, 2011) was not adequate to obtain positive results. 

In this study we performed RT-PCR using the most sensitive primer pair that was recommended by the NCPN-R. The RRD monitoring network (roserosette.org) is a citizen-scientist approach to reporting RRD and providing samples to USA university diagnostic laboratories, which typically employ an RT-PCR test using the RRVF/R primers [7,8,26,29]. Because we reported primer sets 3 and 4 for RT-RPA detection of RRV in *N. benthamiana*, we tested the reliability of these with two other diagnostic primer sets that were reported for diagnostic RT-PCR assays. Our goal was to employ a reliable primer set in the absence of PCR amplification facilitators. As predicted the RRVF/R primers were the most reliable while the remaining three produced false-negative results. Because the rose tissues can have PCR inhibitory compounds, it has been suggested that including PCR amplification facilitators such as BSA or PVP is necessary to boost success and reduce false negatives [25]. Our observations are also confirmed by another study comparing the amplicon results of nine primer pairs tested on RNA from fifty RRV-infected rose samples [29]. This study also found the RRVF/R primers to be the most sensitive and reliable (Di Bello et al. (2018). These primers are routinely employed by the National Clean Plant Diagnostic Network-Roses (NCPDN-R) for national monitoring of RRV in the environment [6,8,27]. 

Seventeen plant species were inoculated with RRV-containing plant sap and all but one produced positive RT-PCR results across a period of 5 wpi. Undoubtedly, the terminal leaves of some plants tested positive for RRV, and in the following week, the newly emerging terminal leaves tested negative. These data support a hypothesis that RRV spreads slowly into new emerging leaves. These data also indicate that repeated sampling over several weeks is necessary to accurately confirm infection. Given the evidence presented in Figure 2 in which leaves of the same rose plant can test positive and negative for RRV, we surmised that regardless of the host, RRV may not be uniformly spreading throughout the plant, and repeated sampling may be necessary to detect infection. Other studies also reported evidence that RRV disease symptoms are not uniform across the plant; virus titers can be below the detection limits of the assays; or RT-PCRs produce false negative results due to PCR inhibitors in the nucleic acid extracts [29,30,31].

The patterns of virus symptoms and detection in the apical tissues across plant species led us to consider that different plant species may display various degrees of resistance, tolerance, or susceptibility [26]. If RRV were a pathogen of these secondary hosts, then the disease should influence host fitness and would also be impacted by host defenses to limit infection. For these reasons, Arabidopsis and lupins are likely non-hosts for RRV since RT-PCR diagnostics showed no amplicons throughout 5 wpi. The necrotic lesions on spinach leaves might possibly be attributable to a hypersensitive defense response to limit virus infection. It is reasonable to then consider the negative RT-PCR results in the upper leaves at 2, 3, and 4 wpi as evidence supporting this hypothesis. In this case, the positive RT-PCR data obtained at 5 wpi would suggest that RRV eventually evaded the host defenses to produce systemic infection. Further experiments are needed to understand the genetic basis of RRV-spinach interactions. 

The pattern of systemic RRV accumulation across the remaining 13 plants, as demonstrated by RT-PCR, likely reflects slow movement or low titers in the upper leaves rather than a common genetic mechanism of resistance. RRV disease was most obvious in peanut and cucumber plants as represented by significant leaf deformations. Other plants showed chlorotic spots or flecks on the upper leaves. Overall, the pattern of infection across plant species does not support a model for host recovery from infection, which is characterized by an initial severe disease and progressively decreasing or disappearance of symptoms in the terminal leaves. Individual yellow squash, *N. glutinosa*, and cucumber plants which tested positive for RRV at 2 wpi but negative at later weeks might represent examples of recovery from infection, although these plants never showed the severe disease or reduction in symptoms. These data preferably support a model of a slowly spreading virus.

Tolerant hosts usually show reduced virus levels and symptoms that persist throughout the lifespan of the plant. Most of the plant species tested represent a non-recovery virus accommodation where infection persists at low levels. Plant growth did not appear to be influenced by RRV infection, another feature of genetic tolerance to infection. It is unlikely that tolerance to RRV would be associated with an immunological event because there is little change in the prevalence of RRV. In general, RRV does not seem to cause mortality or impact fitness of these herbaceous hosts. The disease phenotypes and RT-PCR results suggest that RRV has a low capacity for a virulence relationship with these hosts. 

## Figures and Tables

**Table 1:** Plants used for RRV inoculation study.

Plant Family	Plant Name	Scientific Name	Cultivar or Type
*Cucurbitaceae*	Cucumber	*Cucumis sativus*	Harris
	Yellow squash	*Cucurbita pepo var. recticollis*	yellow crookneck squash
	Zucchini	*Cucurbita pepo*	dark green zucchini
	Winter squash	*Cucurbita maxima*	Butter cup
	Pepper	*Capsicum annuum*	California Wonder
	Tomato	*Solanum lycopersicum*	Brandywine heirloom
*Solanaceae*	Tobacco	*N. rustica*	N/A *
	Tobacco	*N. benthamiana*	N/A
	Tobacco	*N. glutinosa*	N/A
	Soybean	*Glycine max*	Uidori giant
*Leguminosae*	Pea	*Pisum sativum*	Lincoln
	Lupine	*Lupinus* sp.	N/A
*Malvaceae*	Okra	*Abelmoschus esculentus*	Clemson Spineless
*Amaranthaceae*	Spinach	*Spinacia oleracea*	Bloomshade Long Standing
	Goosefoot	*Chenopodium amaranticolor*	N/A
	Quinoa	*C. quinoa*	N/A
*Brassicaceae*	Thale cress	*Arabidopsis thaliana*	Col-0

* N/A, not available.

**Table 2:** List of primers used for *Rose rosette virus* detection by RT-PCR.

Primer Set:	Name	Sequence 5′ to 3′	Product Size (nt)	Position	Target Gene	Reference
1	RRVF RRVR	GCACATCCAACACTCTTGCAGC CTTATTTGAAGCTGCTCCTTGATTTC	271	154–176 425–399	ORF3 (NP)	[23]
2	RRV-2F RRV-2R	TGCTATAAGTCTCATTGGAAGAGAA CCTATAGCTTCATCATTCCTCTTTG	104	881–906 986–961	ORF3 (NP)	[25]
3	RRV3F2 RRV30917R	GGCATAGCTGTTTCTTATCTTTCTAGG AGGGCGAATTCTTCTCTTCC	551	366–393 917–897	ORF3 (NP)	[13]
4	agRRV4-F1 agRRV4-R1	AAACTCAATCTACAGCTGGATTCAT GTCCATCTCTTGAGGGATATTTTCAG	500	636–661 1136–1110	ORF4 (MP)	[13]
5	RmActin-F RmActin-R	AGGGTTTGCTGGAGATGATG CGGGTTAAGAGGTGCTTCAG	280		Actin *	This study

* RmActin-F/R primers correspond to the sequence provided in NCBI, GenBank ID: JN399226.1.

**Table 3:** Slow systemic spread of RRV in herbaceous hosts verified using two-step RT-PCR *.

Plant	2 wpi	3 wpi	4 wpi	5 wpi	Total †	Mock
Cucumber	3	2	2	2	5/5	0/3
Yellow squash	1	0	0	0	1/10	0/3
Winter squash	0	0	3	4	5/5	0/3
Pumpkin	3	-	-	-	3/5	0/3
Pepper	2	2	2	4	4/5	0/3
Tomato	2	2	0	0	3/10	0/3
*N. rustica*	2	3	6	0	7/10	0/3
*N. glutinosa*	2	0	0	-	2/6	0/3
Pea	2	1	0	0	2/6	0/3
Peanut	2	2	3	4	4/6	0/3
Soybean	1	0	0	2	3/10	0/3
Lupine	0	0	0	0	0/5	0/3
Okra	0	2	2	2	3/5	0/3
Spinach	0	0	0	4	4/5	0/3
*C. amaranticolor*	0	0	2	2	3/5	0/3
*C. quinoa*	0	0	2	2	2/5	0/3
*Arabidopsis*	0	0	0	0	0/6	0/3

* Terminal leaves were sampled. RT-PCR-positive plants at 2 or 3 wpi may produce negative newer emerging leaves in the subsequent week(s). However, the leaves that tested positive continued to test positive when resampled. “-” indicates plants were not sampled. wpi = weeks post-inoculation. † The proportion of plants that tested positive across the 5 weeks of the experiment relative to the total number of inoculated plants. PCR amplicons were sequenced and pairwise aligned against RRV reference sequence NC_015300.1 to confirm RRV systemic spread throughout the infected plants.

**Table 4:** RT-PCR results for 2–5 wpi with RRV following *Agrobacterium*-delivered RRV infectious cDNA clone or mechanically inoculated using crude sap from RRV-infected roses.

Plant Type	Infectious cDNA Clone of RRV			Crude Sap from RRV-Infected Rose		
	Local (2–3 wpi)	Systemic (2–3 wpi)	Systemic (4–5 wpi)	Local (2 wpi)	Systemic (2–3 wpi)	Systemic (4–5 wpi)
*N. benthamiana*	5/5	0/5	4/5	5/5	0/5	5/5
Pepper	5/5	0/5	3/5	5/5	2/5	4/5
Spinach	5/5	0/5	2/5	1/5	2/5	4/5
Okra	5/5	0/5	2/5	3/5	0/5	2/5
Zucchini	5/5	0/5	1/5	4/5	2/5	3/5

## Data Availability

Not applicable.
